# Protective Effects of Cyanidin-3-O-Glucoside Against Neurotoxin Acrylamide Through Alleviating Mitochondrial Dysfunction

**DOI:** 10.3390/foods14223826

**Published:** 2025-11-08

**Authors:** Liuqing Yang, Lujia Zhang, Li Dong, Yanli Ma, Lei Zhao, Ruoyang Xu, Fang Chen, Yinghua Luo

**Affiliations:** 1 Zhang Zhongjing School of Chinese Medicine, Nanyang Institute of Technology, Nanyang 473004, China; 3152094@nyist.edu.cn (L.Y.); 17532353622@163.com (R.X.); 2 National Engineering Research Centre for Fruits and Vegetables Processing, Key Laboratory of Fruits and Vegetables Processing, Ministry of Agriculture, Engineering Research Centre for Fruits and Vegetables Processing, Ministry of Education, College of Food Science and Nutritional Engineering, China Agricultural University, Beijing 100083, China

**Keywords:** acrylamide, cyanidin-3-O-glucoside, neurotoxicity, mitochondria

## Abstract

Acrylamide (AA), a well-known neurotoxin, shows obvious damage to the nervous system. Cyanidin-3-O-glucoside (C3G), a representative anthocyanin, is identified as a promising neuroprotective agent as its excellent antioxidant capacity. This study evaluated the mitoprotective effects of C3G against AA-mediated neurotoxicity. The results showed that pretreatment with C3G (10 μmol/L) significantly lessened the reduction in AA-induced cell survival rate, increasing cell viability by 1.31 times compared to the AA-only group. C3G reduced intracellular ROS and MDA level accumulation by 84.0% and 61.9%, respectively. Furthermore, C3G suppressed the activation of NLRP3 inflammasome and Caspase-3-dependent apoptosis pathways induced by AA. Further mitochondrial analysis revealed that C3G pretreatment enhanced mitochondrial membrane potential recovery by 1.50 times and preserved the mitochondrial ultrastructure, while also restoring the aerobic respiratory capacity. PCR array demonstrated that C3G reversed the AA-induced downregulation of mitochondrial biogenesis genes PGC-1α and TFAM by 2.67-fold and 1.88-fold, respectively, and mitochondrial dynamics genes Mfn2 and Opa1 by 2.76-fold and 3.08-fold. Further in vivo studies confirmed that the blueberry anthocyanin extracts, which are mainly composed of C3G, showed neuroprotective function through maintaining mitochondrial function, alleviating inflammation, and apoptosis. This article provides new insights into the neuroprotective effects of C3G.

## 1. Introduction

Acrylamide (AA; CAS Reg. 79-06-1) is a highly hydrophilic, colorless, odorless, crystalline powder. In the chemical industry, it serves as a key monomer for synthesizing polymers and gels that are extensively applied in cosmetics, paper production, textiles, and wastewater treatment [1,2]. AA has also been reported to exist in soil around the factory, drinking water, and even cigarette smoke [3]. Furthermore, a 2002 report indicated that humans are exposed to significant dietary AA from high-temperature (>120 °C) cooking of high-carbohydrate foods, including baked goods (biscuits and bread) and starchy processed foods (fried potatoes and breakfast cereals) [4,5,6]. Emerging evidence increasingly indicates that acrylamide (AA) exerts multifaceted health risks in animals, encompassing neurotoxicity, developmental toxicity, liver toxicity, reproductive toxicity, and carcinogenicity [7,8]. Considering the environmental and dietary exposure of AA could be a potential threat to human health, looking for toxicity intervention mechanisms to avoid the damage of AA is a very worthwhile research topic.

Notably, AA is most recognized as a potent neurotoxin in humans and animals [9]. Although the precise molecular mechanism underlying AA neurotoxicity remains not completely understood, the destruction of cellular redox balance by AA has been considered as a potential reason that causes the neurotoxicity [10,11]. By disrupting cellular redox balance, overproduction of reactive oxygen species (ROS) acts as a key trigger to initiate both inflammatory responses and cellular apoptosis in biological systems [12]. In mammalian cells, mitochondria function as major intracellular sources of ROS [13]. Previous studies have shown that AA can induce mitochondrial dysfunction in two common glial cell models: human astrocytoma cells and BV-2 microglial cells [14,15]. Our previous studies indicated that AA not only destroyed redox balance and damaged mitochondrial structures but also decreased the expression of mitochondrial biogenesis-related genes peroxisome proliferator activator receptor gamma coactivator-1α (PGC-1α) and mitochondrial transcription factor A (TFAM), as well as the dynamics-related genes Mfn2 and Opa1 [16]. Moreover, the activation of excessive apoptosis and inflammatory pathways is considered to be the main downstream signaling pathway of nerve damage induced by AA [17]. Therefore, searching for natural active substances that can target and protect mitochondrial function may be an excellent choice for intervening in AA neurotoxicity.

Anthocyanins are a kind of polyphenol found in nature, which are considered to be the principal bioactive compounds present in most berries, vegetables, and beans [18,19]. Anthocyanins are associated with health enhancement properties as their protective function in the amelioration of oxidative stress [20]. Pharmacokinetic studies demonstrate that anthocyanins can be absorbed as intact glycosides, cross the blood–brain barrier, and exhibit a widespread distribution among various brain regions [21,22]. Cyanidin-3-O-glucoside (C3G), the most abundant anthocyanin, exhibits free radical scavenging capacity, modulates reductase activity, and possesses anti-inflammatory and anti-apoptotic properties [23,24]. Neuronal limited regeneration underlies central nervous system (CNS) vulnerability to oxidative stress [25]. While numerous studies report that C3G can attenuate oxidative damage, apoptosis, and inflammation in the CNS to indicate the neuroprotective effects [26,27]. Previous studies reported that anthocyanin extracts can reverse liver mitochondrial dysfunction and alleviate apoptosis caused by AA in the liver and inhibit the inflammatory response. Song reported that C3G exhibited excellent ability to attenuate AA-induced oxidative damage to genomic DNA in MDA-MB-231 cells [28]. Nevertheless, whether C3G can confer protective potential in AA-induced impairments of mitochondrial biogenesis and dynamics remains incompletely characterized. This study investigated the protective role of C3G on mitochondria against AA-induced neurotoxicity from cell culture to animal models.

As the most diverse neuroglia in the CNS, astrocytes regulate its homeostatic microenvironment [29]. Impairment of astrocyte function is a key factor in neurodegeneration and can impede neuronal recovery [30]. Although the neuroprotective effect of anthocyanins against AA has been reported, there is still a lack of research on the protective mechanism of anthocyanins on glial cells at the mitochondrial level. Research concerning the neurotoxicity of AA predominantly centers on neuronal damage, while largely neglecting the roles of other neural cell types, including astrocytes and microglia, in responding to AA toxicity. In the present study, we systematically explored the protective effects of C3G, a representative anthocyanin, against mitochondrial structural and functional impairment induced by AA treatment in vitro. To validate these findings, we further conducted in vivo experiments using blueberry anthocyanin extract (BAE) administered via intragastric gavage, which contains high levels of C3G as previously characterized [31]. The purpose of this study was to determine whether C3G can confer protective effects on mitochondria, thereby mitigating the neuronal damage elicited by AA exposure.

## 2. Materials and Methods

### 2.1. Chemicals

Acrylamide was obtained from Sigma (Oakville, ON, Canada). Cyanidin-3-O-glucoside (purity > 97%), thiazolyl blue tetrazolium bromide (MTT), 2′,7′-dichlorofluorescein diacetate (DCFH-DA), and 5,5′,6,6′-tetrachloro-1,1′,3,3′-tetraethylbenzimidazolyl carbocyanine iodide (JC-1) were purchased from Beijing Solarbio Science and Technology Co., Ltd.(Beijing, China). Culture medium DMEM/F12, fetal bovine serum (FBS), phosphate-buffered saline (PBS, pH 7.4), 0.25% Trypsin-EDTA, and penicillin/streptomycin (10,000 U/mL) were acquired from Gibco (Thermo Fisher Scientific Inc., Rochester, NY, USA). Reduced glutathione (GSH) and malondialdehyde (MDA) assay kits were supplied by Nanjing Jiancheng Bioengineering Institute (Nanjing, China). Seahorse XF assay medium and Seahorse XF calibrant solution were purchased from Agilent Technologies (Santa Clara, CA, USA). BAE was donated by Daxinganling Lingonberry Boreal Biotech Co., Ltd. (Jiagedaqi, China), and the components were tested as reported in the previous article, which indicated that C3G accounted for 70% [32].

### 2.2. Cell and Animal Treatment

**Figure 1:**
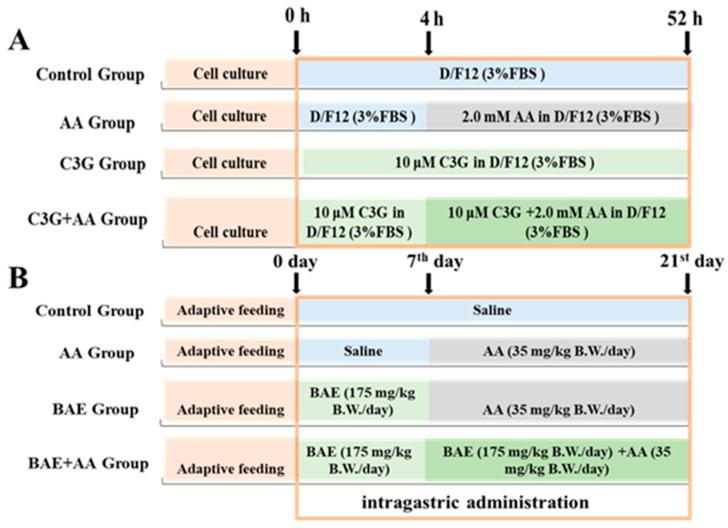
In vivo and in vitro experimental grouping. (**A**). Cell treatment protocol. (**B**). Animal treatment protocol.

Primary astrocyte cultures, isolated from the brains of newborn Sprague-Dawley (SD) rats, were prepared with reference to a previously established protocol [16]. For the MTT assay, astrocytes were pretreated with increasing concentrations of C3G (0, 5, 10, 20, 40, and 80 μmol/L) for 4 h, and then the medium was changed to 2.0 mmol/L AA for another 48 h. For another experiment, cells were divided into 4 groups, and the processing of each group is shown in the following Figure 1A.

A total of twenty-four 7-week-old female SD rats were purchased from Beijing Vital River Laboratory Animal Technology Co., Ltd. (Beijing, China; Certificate NO.SCXK Jing 2022-0052) and were raised in SPF SPF-grade environment with adequate clean water and standard food. After one week of acclimatization, rats were randomly divided into 4 groups (*n* = 6) and the treatments were described as shown in Figure 1B. No unintended mortality or signs of severe distress occurred during the administration period. Following 3 weeks of intervention, rats were euthanized by gradual-fill carbon dioxide inhalation in accordance with AVMA guidelines. Death was confirmed by cessation of heartbeat and respiration for >5 min. Then the brain tissues were obtained and stored at −80 °C until analysis. This method was approved by China Agricultural University Laboratory Animal Welfare and Animal Experimental Ethical Committee (No.AW70401202-4-1). All molecular assays were performed on biological replicates derived from all 6 animals per group to account for individual variability and ensure conclusions were drawn from population-level trends.

### 2.3. MTT Assay

Cell viability was performed by MTT assay. Briefly, after treatment with the reagent, astrocytes were incubated with MTT solution (0.5 mg/mL) at 37 °C for 4 h. The medium was then removed, and 100 μL of DMSO was added to each well. Plates were agitated for 5 min, and the optical density was measured by a microplate reader (Tecan, Männedorf, Switzerland).

### 2.4. Measurement of ROS Generation

Following reagent specifications, intracellular ROS levels were assessed. After preparation, cells were imaged on a fluorescence microscope (Zeiss Observer A1, Oberkochen, Germany) and final fluorescence readings (λ_ex_ = 488 nm, λ_em_ = 525 nm) were acquired by a microplate reader.

### 2.5. Measurement of GSH Levels and MDA Content

Reduced GSH levels and MDA content in astrocytes and brain tissues were measured using commercially available assay kits following the manufacturer’s protocols.

### 2.6. Mitochondrial Membrane Potential Assay

Mitochondrial membrane potential (MMP) was assessed using the JC-1 assay kit according to the manufacturer’s protocol.

### 2.7. Mitochondrial ROS Detection

Mitochondrial ROS (mtROS) levels were assessed using the MitoSOX™ Red fluorescent probe (5.0 μM), which specifically targets superoxide within mitochondria [33]. After treatment, cells were then analyzed by flow cytometry (BD Accuri C6, San Diego, CA, USA). MtROS-positive cells were quantified based on fluorescence intensity (excitation/emission = 510/580 nm).

### 2.8. AO/EB Double Staining

Apoptotic cells were observed by AO and EB double staining according to the instructions. The fluorescence imaging analysis was visualized and captured with the inverted fluorescence microscope (Zeiss Observer A1).

### 2.9. Extracellular Flux Analyses

Mitochondrial function was assessed by measuring oxygen consumption rates (OCRs) using the Seahorse XF24 Extracellular Flux Analyzer (Agilent Technologies, Santa Clara, CA, USA). OCR measurements were performed following the manufacturer’s mitochondrial stress test protocol [16].

### 2.10. Quantitative Real-Time PCR

The extraction as well as reverse transcription process of RNA from cells or brains was followed by the manufacturer’s instructions (Takara Bio, Kyoto, Japan). Quantitative real-time PCR (qRT-PCR) was performed using the LightCycler 480 system (Roche, Basel, Switzerland) to measure the mRNA expression levels of target genes listed in Appendix A.1. The relative expression levels of each gene were conducted by the 2^−ΔΔCT^ method based on GAPDH as a reference gene.

### 2.11. Western Blot Analysis

Proteins were extracted from both cells and brain tissues with RIPA lysis buffer (Thermo Fisher, MA, USA), and the concentration of protein was quantified by the BCA method. The detailed procedure for Western blotting was performed as described previously [34].

### 2.12. Statistical Analysis

All experiments were performed in triplicate. Data are expressed as mean ± standard deviation (SD) and analyzed via one-way ANOVA with Tukey’s post hoc test for multiple comparisons using GraphPad Prism (Version 9.5; GraphPad Software, San Diego, CA, USA). Statistical significance was set at *p* < 0.05.

## 3. Results

### 3.1. C3G Alleviates AA-Induced Cytotoxicity and Oxidative Stress

**Figure 2:**
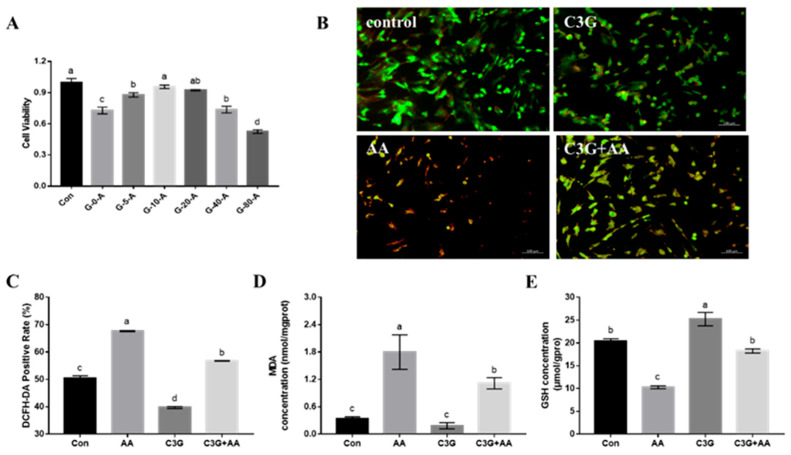
Effects of C3G on AA-induced cytotoxicity and oxidative stress. (**A**) Cell survival rate; (**B**) representative images of AO/EB staining; (**C**) ROS detections; (**D**) MDA content; (**E**) reduced GSH content. Different letters denote significant differences (*p* < 0.05) among treatment groups.

As the most common and representative anthocyanin, C3G was selected to assess its protective effects against AA’s neurotoxicity [24]. The C3G pretreatment concentration (5–100 μmol/L) was applied based on prior studies [35] and the pilot experiment. This range ensured the concentration was non-cytotoxic while providing protection against subsequent AA challenge. As shown in Figure 2A, astrocytes pre-treated with 10 μmol/L C3G for 4 h, followed by 48 h exposure to 2.0 mmol/L AA, exhibited the greatest protective effect. In contrast, concentrations of C3G exceeding 40 μmol/L resulted in lower cell viability compared to the AA treatment group alone. Accordingly, we chose 10 μmol/L of C3G as the treatment concentration for subsequent experiments. AO/EB staining was then applied to verify that C3G can better protect against cell damage caused by AA. Compared to the AA group, the C3G + AA group showed fewer EB-positive cells in Figure 2B. The modulatory impact of C3G on oxidative stress was further analyzed. Exposure of astrocytes to AA resulted in a marked elevation of intracellular ROS and MDA levels. Pretreatment with C3G significantly reduces (*p* < 0.05) the ROS accumulation and MDA content induced by AA. To compare the antioxidant capacity of cells, intracellular GSH levels were further examined. Compared with the AA alone-treated group, the GSH content in the C3G and AA co-treated group increased by 1.78 times, and reverted to control levels (Figure 2E). In summary, C3G treatment effectively attenuated AA-induced intracellular oxidative damage.

### 3.2. C3G Suppresses AA-Induced Damage to Mitochondrial Structure, Redox Balance, and Respiratory Function

**Figure 3:**
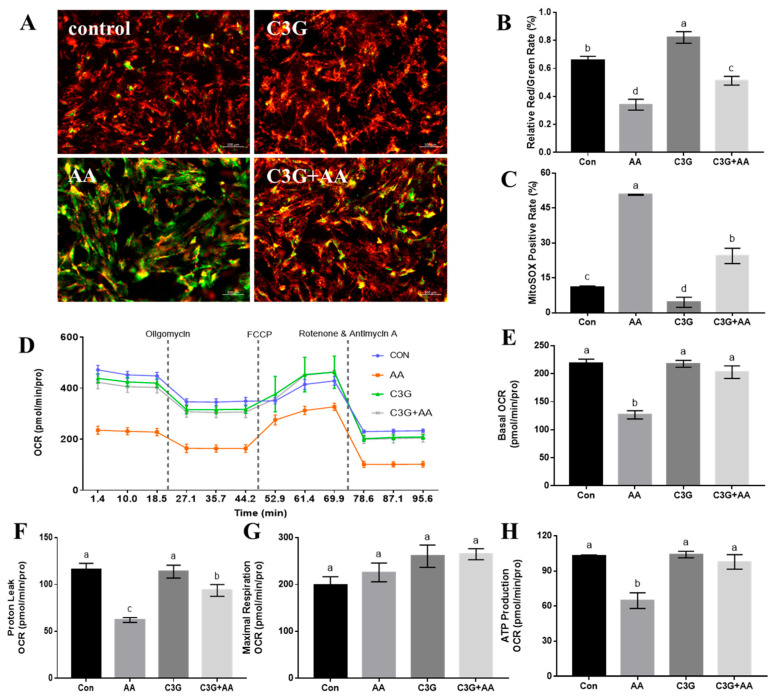
The protective effect of C3G on mitochondrial function. (**A**) Representative pictures of JC-1 staining; (**B**) quantification of fluorescence intensity of JC-1 staining; (**C**) detection of mitochondrial ROS; (**D**) real-time aerobic respiration rate of cells; (**E**) basic respiration rate; (**F**) proton leakage; (**G**) maximum respiration rate; (**H**) ATP production capacity. Different letters denote significant differences (*p* < 0.05) among treatment groups.

It has been confirmed that AA could induce mitochondrial dysfunction by disrupting the mitochondrial structure; hence, we explored whether C3G can restore mitochondrial function after AA exposure in primary astrocytes. MMP is commonly considered an indicator of mitochondria. Compared with the control group, AA decreased the JC-1 aggregation to JC-1 monomer (red/green fluorescence) ratio, indicating a reduced MMP in AA-treated cells. While C3G pretreatment can increase the intensity of red fluorescence, showing the protective effect of AA-induced decreases in MMP (Figure 3A,B). We then elevated the mtROS levels in different treatment groups (Figure 3C). C3G pretreatment significantly lowered the mtROS-positive rate relative to the AA group (*p* < 0.05). Furthermore, the C3G-only treatment group exhibited lower mtROS-positive levels than the control group. These findings suggest that C3G intervention reduced mtROS production and attenuated the decline in MMP induced by AA.

Next, the aerobic respiration rate of cells with different treatments was monitored in real-time, in order to evaluate whether C3G pretreatment could help resume the function of mitochondria after AA exposure. The results indicate that C3G intervention can reverse the decrease in cellular aerobic respiration level caused by AA treatment (Figure 3D), fully restore cellular basal respiration (Figure 3E), proton leakage (Figure 3F), and ATP production levels to normal levels (Figure 3H), and ensure the normal operation of aerobic respiration function. Through the mitigation of AA-induced damage to mitochondrial aerobic respiration, C3G effectively rescues mitochondrial function and thereby achieves cytoprotection.

### 3.3. C3G Ameliorates AA-Caused Mitochondrial Function-Related Gene Expression Abnormalities

**Figure 4:**
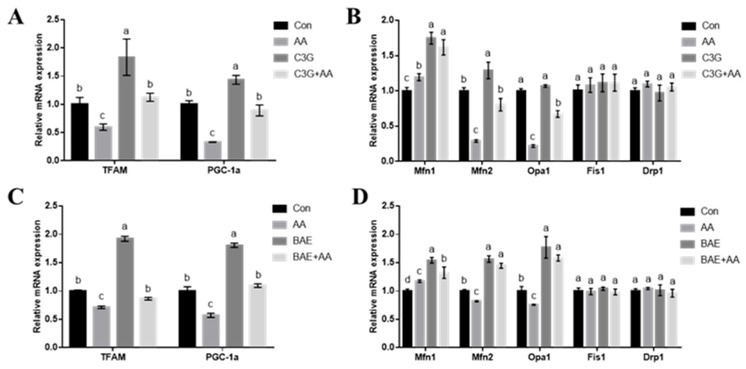
Intervention of C3G/BAE on AA-induced mitochondrial-related gene expression disorder. (**A**) Mitochondrial biosynthesis-related gene expression in astrocytes; (**B**) mitochondrial dynamics-related gene expression in astrocytes; (**C**) mitochondrial biosynthesis-related gene expression in the brain; (**D**) mitochondrial dynamics-related gene expression in the brain. Different letters denote significant differences (*p* < 0.05) among treatment groups.

Previous studies have shown that AA impairs mitochondrial biogenesis and dynamics-related gene expression, leading to mitochondrial dysfunction [16]. Accordingly, we evaluated how C3G protects the transcriptional expression levels of key genes, including TFAM and PGC-1α (biogenesis), as well as Mfn1, Mfn2, Opa1, Fis1, and Drp1 (dynamics). Figure 4A shows that AA treatment led to downregulation of PGC-1α and TFAM expression versus the control group (*p* < 0.05), C3G and AA co-treatment reversed this decrease. Similarly, the suppression of the fusion genes Mfn2 and Opa1 by AA was also reversed upon C3G co-treatment. C3G monotherapy markedly enhanced Mfn1 levels (*p* < 0.05). However, neither AA nor C3G treatment significantly affected the expression of mitochondrial fission regulators Drp1 and Fis1. In summary, it can be seen that C3G treatment can indeed restore gene expression related to mitochondrial biogenesis and mitochondrial fusion, thereby showing a protective role against AA-induced mitochondrial functional damage.

Anthocyanin intervention on AA-disturbed mitochondrial dynamics and biogenesis was further verified in rodent models. Transcript levels of mitochondrial functional regulators were assessed in rat brains. Consistent with the in vitro findings in astrocytes, the transcription levels of PGC-1α and TFAM (Figure 4C), as well as mitochondrial fusion-related genes Mfn2 and Opa1 (Figure 4D), were restored to a level comparable to the control group, indicating that BAE treatment can relieve the expression disorder of mitochondrial function-related genes caused by AA. Interestingly, in the BAE alone treatment group, significant upregulation of TFAM and PGC-1α expression was observed, suggesting an intrinsic activating effect of anthocyanins on mitochondrial biogenesis.

### 3.4. C3G Mitigates AA-Induced Apoptosis and Inflammatory Response

**Figure 5:**
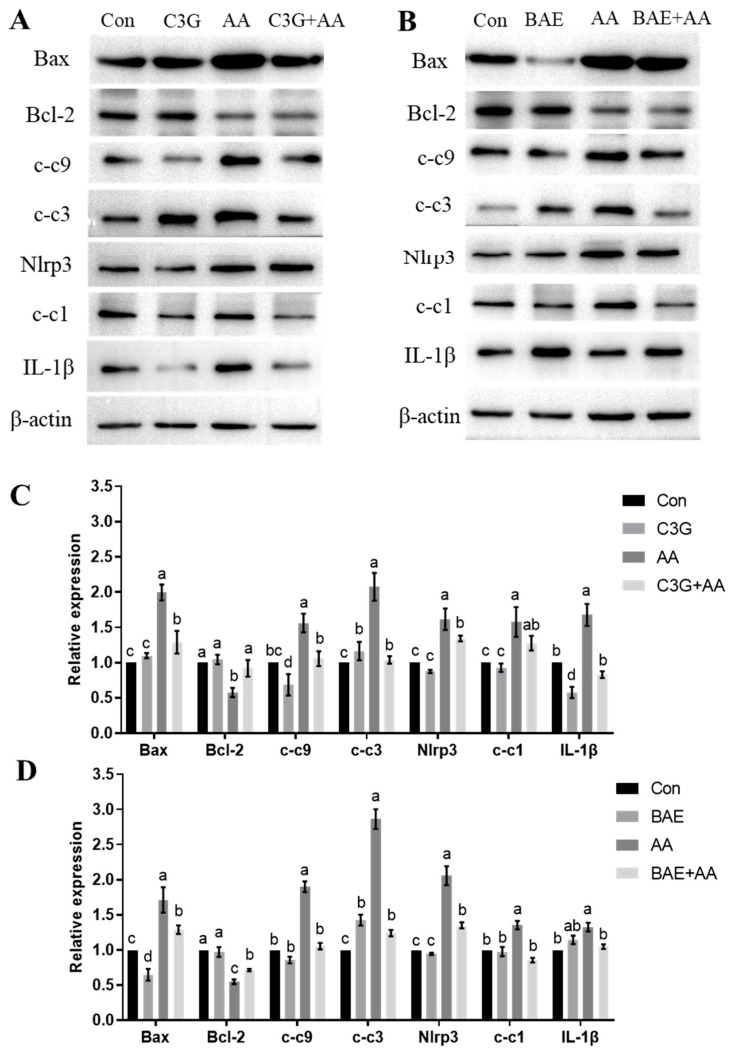
The effect of C3G/BAE intervention on apoptosis and the NLRP3 pathway induced by AA. (**A**) Representative immunoblots of apoptosis-related proteins and the NLRP3 pathway in cell experiments; (**B**) representative immunoblots of apoptosis-related proteins and the NLRP3 pathway in animal experiments; (**C**) quantification of the immunoblots in A; (**D**) quantification of the immunoblots in B. Different letters denote significant differences (*p* < 0.05) among treatment groups.

The consequences of mitochondrial dysfunction mainly involve caspase-3-induced apoptosis and NLRP3 inflammasome activation, leading to cellular damage [34,36]. We then investigated whether anthocyanins pretreatment blocks apoptosis and inflammatory response induced by AA across organismal and cellular levels. As shown in Figure 5A, treatment with AA led to a significant upregulation in the expression of apoptosis-related proteins Bax, cleaved-caspase-9 (c-c9), and cleaved-caspase-3 (c-c3), accompanied by downregulation of the anti-apoptotic protein Bcl-2. Meanwhile, compared with the control group, AA-intoxicated astrocytes exhibited marked increases in NLRP3, cleaved-caspase-1 (c-c1), and the downstream inflammatory factor IL-1β. However, supplementation with C3G helped reduce these changes compared to AA treatment alone. As demonstrated by WB analysis of the in vivo results (Figure 5B), administration of AA alone significantly upregulated the expression of pro-apoptotic proteins (Bax, c-c9, and c-c3) as well as NLRP3 inflammasome-associated factors (NLRP3, c-c1, and IL-1β) compared to the control group (*p* < 0.05). Notably, co-treatment with BAE and AA partially mitigated this AA-induced protein overexpression, resulting in reduced levels of these pro-inflammatory and pro-apoptotic markers. In contrast to the cell-based findings, however, we observed a slight but statistically significant increase in inflammatory markers—specifically elevated IL-1β levels—in BAE-exposed rat brains versus controls. This elevation may suggest activation of the immune system by BAE. These experimental findings indicate that anthocyanins demonstrate neuroprotective effects against AA-induced nerve damage, as evidenced by their efficacy across cellular and organismal levels.

## 4. Discussion

AA, a recognized neurotoxin, has been reported to cause neurological disorders, including cognitive dysfunction and neuronal impairment [37]. Given the persistent risk of AA exposure in daily human life, dietary nutritional intervention emerges as a promising strategy to mitigate its associated adverse health effects. Since the neuroprotective effects of anthocyanins on the mitochondrial level have not been extensively investigated, this study provides definitive evidence that anthocyanins exert mitochondrial protection against AA-induced neurotoxicity, as demonstrated by maintaining normal mitochondrial aerobic respiration, ensuring proper expression of mitochondrial-related genes, and mitigating cellular apoptosis and inflammatory responses.

The neurotoxicity of AA is primarily mediated by oxidative stress-initiated cascades, including depletion of antioxidants and accumulation of peroxides. It is confirmed that AA could cause the increasement of ROS in cells, while the excessive ROS triggered noteworthy oxidative damages in the brain and further caused the decrease in antioxidant enzyme activity (SOD and CAT) and GSH levels [38]. Moreover, AA intoxication elevated the MDA and NO levels in testicles and cerebellum [39,40]. Consistent with this, Yan et al. reported that AA decreased GSH levels while increasing MDA and ROS levels in both SH-SY5Y cells and rat striatum [41]. Mirroring prior reports, astrocytic systems demonstrated elevated ROS and MDA accumulation paired with reduced GSH reserves after AA treatment. Intake of 25 g/day blueberry-derived anthocyanins (269 mg, C3G-equivalents) potentiated neuronal responses in the aging cohort [42]. Given the reported ability of anthocyanins and their extracts to mitigate CNS damage caused by environmental toxins such as bisphenol A [43], perfluorooctanoic acid [44], and aluminum chloride [45], we characterized C3G-mediated cytoprotection against AA-induced redox imbalance in astroglial cells.

Studies have confirmed that C3G can attenuate neurotoxicity induced by H_2_O_2_ [46], ethanol [47], LPS [48], as well as AA [28] in vitro. Our research also confirmed the good protective effects of C3G against AA in primary astrocytes, specifically manifested as increased cell viability and decreased levels of oxidative stress. In animal studies on C3G, administration doses ranging from 7.2 mg/kg to 100 mg/kg or higher have demonstrated efficacy against obesity-induced oxidative stress, inflammation, liver injury, alcohol-induced liver injury (ALD), and Alzheimer’s disease (AD). Oral C3G administration (50 mg/kg, 8 weeks) to high-fat-diet (HFD) fed mice reduced hepatic and plasma triglycerides, decreased adiposity, increased glucose tolerance, and shifted hepatic metabolism [49]. Intragastric gavage for 6 weeks, 80 mg/kg C3G nanoparticles (purity ≥ 98%), ameliorated fat accumulation and liver oxidative stress induced by HFD [50]. In male C57BL/6J mice, 11-week C3G supplementation (7.2 mg/kg/day) attenuated HFHS diet-induced metabolic dysregulation, inflammatory responses, and gut microbiota perturbations, revealing prebiotic efficacy [51]. By modulating enteric microbial ecology and associated metabolites, C3G (100 mg/kg/d) rescued alcohol-mediated hepatic damage in murine ALD models, affirming its functional food value [52]. In APPswe AD mice, 16-week oral administration of 30 mg/kg/day C3G ameliorated cognitive deficits and AD pathology by facilitating Aβ clearance, quenching neuroinflammation, augmenting endogenous antioxidants, and attenuating pathogenic tau phosphorylation [27].

Mitochondria play vital functions in providing energy for cells and organs, promoting organ recovery, as well as maintaining homeostasis. Recent investigations have offered strong relationships between the neurotoxic effects of AA and mitochondrial dysfunction [16]. Mitochondrial dysfunction culminates in a cascade of detrimental events, including ROS accumulation, a decrease in MMP, and dysfunction of the respiratory chain [53]. In the current study, AA significantly decreased Bcl-2 and increased Bax expression, revealing the loss of mitochondrial membrane structure, while C3G reversed the mitochondrial structural damage caused by AA. The normal functioning of the oxidative phosphorylation system is required to generate energy, and the Seahorse XF24 Extracellular Flux Analyzer was applied to evaluate cellular respiration under different treatments. Similarly to the literature [54], our study provides evidence that AA induced mitochondrial respiratory dysfunction, while C3G treatment can help astrocytes restore normal respiratory status and maintain normal mitochondrial function. Next, we further evaluated the destructive effect of AA on cellular mitochondria and the protective effect of anthocyanins at the genetic level.

The environmental stimuli affect the normal function of mitochondria by interfering with their quantity, distribution, and morphology, especially in the process of mitochondrial biogenesis and dynamics [55]. Mitochondrial biogenesis in the brain is regulated by PGC-1α and transcriptional regulators TFAM, which drives transcription and replication of mtDNA [56]. PGC-1α orchestrates nuclear-mitochondrial communication by initiating a transcriptional cascade that activates TFAM, thereby coupling nuclear regulatory programs with mitochondrial biogenesis [57]. AA exposure systemically disrupted mitochondrial biogenesis machinery, manifested as significant downregulation of transcriptional coactivator PGC-1α and its target TFAM in both brain tissues and astrocytes. C3G/BAE treatment attenuated this downregulation and restored levels to normal. Mitochondrial dynamics, encompassing fusion and fission, regulate mitochondrial morphology, distribution, and activity [58].In mammals, Mfn1 and Mfn2 are regarded as two main mitofusin homologs, and the lack of Mfn1 and Mfn2 resulted in poor mitochondrial function [59]. Furthermore, OPA1 mediates mitochondrial fusion and is crucial for cristae integrity; its loss leads to severe cristae structural defects [60]. As core components of the mitochondrial fission machinery, Drp1 initiates fission, a process facilitated by Fis1, and targets Drp1 to the mitochondrial outer membrane [61,62]. Our results demonstrate that regulators of mitochondrial fusion (Mfn2/OPA1) were transcriptionally silenced by AA, triggering dynamic collapse and dysfunction, which aligns with the study by Qiang [57]. Previous studies reported that mutations in human Mfn2 or OPA1 were related to neurodegenerative diseases [63]. C3G intervention shows restoration of mitochondrial fission-related gene expression (Mfn2 and OPA1), indicating the protective effect of the neurotoxicity of AA. Similar conclusions have also been confirmed in subsequent animal experiments, and the BAE also showed mitochondrial protection properties in animal experiments. In summary, anthocyanins have shown good protective effects on AA-damaged mitochondria, including maintaining normal mitochondrial function, biogenesis, and dynamic protection. The protective action of C3G on mitochondria has been studied for the first time in primary astrocytes.

There is a close relationship between mitochondria and apoptosis and inflammation. The release of mtROS from damaged mitochondria can trigger caspase-3-dependent apoptosis and activate NLRP3 inflammasomes [64]. Moreover, the depletion of mitofusins, especially OPA1, enhances the susceptibility to apoptotic stimuli [61]. Accumulating evidence suggests that AA induces cellular damage through upregulation of apoptosis-related proteins and pro-inflammatory cytokines, as demonstrated in prior studies [65]. Otherwise, the NLRP3-related inflammatory pathway is activated and involved in AA-induced neuroinflammation. Similarly to the literature, our study provides evidence that AA induced progressive caspase-3-related apoptosis and NLRP3-related inflammation, demonstrating the close relationship between mitochondrial damage caused by AA and subsequent activation of apoptotic and inflammatory pathways. Furthermore, anthocyanins, known to restore mitochondrial function and structure, also reduced AA-induced cell apoptosis and inflammatory response at multiple biological hierarchies. This study breaks new ground by elucidating the specific role of C3G in mitigating AA-induced neurotoxicity through mitochondrial protection. Unlike previous research that primarily focused on general antioxidant properties of anthocyanins, our work pinpointed C3G’s direct suppressive effect on AA-triggered mitochondrial dysfunction, offering a previously unexplored molecular perspective. Moreover, we propose a practical and preventive approach against environmental neurotoxins—dietary supplementation with anthocyanin-rich foods. This approach reorients the paradigm from pharmaceutical interventions toward accessible, nutrition-based solutions, highlighting the neuroprotective potential of the daily diet.

## 5. Conclusions

In conclusion, this study demonstrates that C3G confers significant protection against AA-induced neurotoxicity, with the underlying mechanism involving the maintenance of mitochondrial dynamics and functional homeostasis. Our in vitro findings establish that C3G pretreatment effectively mitigates key hallmarks of AA-induced neuronal damage, including the reduction in cell viability, accumulation of intracellular superoxide, and the activation of both NLRP3-mediated inflammatory and Caspase-3-dependent apoptotic pathways. Crucially, we identified that these protective effects are rooted in C3G’s ability to sustain mitochondrial health. C3G was shown to prevent AA-induced ultrastructural damage to mitochondria and rescue the impairment of aerobic respiration. At the molecular level, PCR array analysis revealed that C3G counteracts the AA-suppressed expression of critical genes governing mitochondrial biogenesis (PGC-1α and TFAM) and dynamics (Mfn2 and Opa1), thereby promoting a healthy mitochondrial network. These findings were strongly supported by our in vivo experiments, which confirmed that BAE, predominantly composed of C3G, exhibits potent neuroprotective activity. The in vitro mechanism was consistent with the protective phenotype observed in vivo, as it maintains mitochondrial function and subsequently alleviates neuroinflammation and apoptosis.

Collectively, the current study offers compelling evidence positioning mitochondrial dysfunction as a critical target in AA-induced neurotoxicity and establishes C3G as a promising therapeutic agent capable of rescuing mitochondrial homeostasis. It provides an effective intervention strategy against neurotoxins through dietary supplements with anthocyanin-enriched foods. Subsequent research will focus on in-depth mechanistic investigations to elucidate how C3G exerts mitochondrial protection and mitigates AA-induced toxicity.

## Data Availability

The data that support the findings of this study are available from the corresponding author upon reasonable request.

## Abbreviations

The following abbreviations are used in this manuscript:

AA	Acrylamide
C3G	Cyanidin-3-O-glucoside
ROS	Reactive oxygen species
PGC-1α	Peroxisome proliferator activator receptor gamma coactivator-1α
TFAM	Mitochondrial transcription factor A
CNS	Central nervous system
BAE	Blueberry anthocyanin extract
MTT	Thiazolyl blue tetrazolium bromide
DCFH-DA	2′,7′-dichlorofluorescein diacetate
JC-1	5,5′,6,6′-tetrachloro-1,1′,3,3′-tetraethylbenzimidazolyl carbocyanine iodide
FBS	Fetal bovine serum
PBS	Phosphate-buffered saline
GSH	Glutathione
MDA	Malondialdehyde
MMP	Mitochondrial membrane potential
mtROS	Mitochondrial ROS
c-c9	Cleaved caspase-9
c-c3	Cleaved caspase-3
c-c1	Cleaved caspase-1

## Appendix A

### Appendix A.1

The primers of qRT-PCR.

**Table A1:** Primers of qRT-PCR.

Gene	Forward (5′-3′)	Reverse (5′-3′)
Mfn1	CAGCAGCAGAGAAGAGGGTTTAT	CAGCACACTGGGGGTAGGAT
Mfn2	AGTGTCAAGACCGTGAACCA	ACACATCAGCATCCAGGCAA
Drp1	GCTAGATGTGCCAGTTCCAGT	TGTGCCATGTCCTCGGATTC
Opa1	GGCACTTCAAGGTCGTCTCA	CACTGCTCTTGGGTCCGATT
Fis1	ACGCCTGCCGTTACTTCTTC	GCAACCCTGCAATCCTTCAC
TFAM	AAATGGCTGAAGTTGGGCGAAGTG	AGCTTCTTGTGCCCAATCCCAATG
PGC-1α	CCGAGAATTCATGGAGCAAT	GTGTGAGGAGGGTCATCGTT
GAPDH	AGTGCCAGCCTCGTCTCATA	GGTAACCAGGCGTCCGATAC

### Appendix A.2

The detailed information about primary antibodies for WB.

**Table A2:** Anti-bodies of WB.

Name	Dilution	Manufacturer	Catalog
Anti-Bcl-2 antibody [E17]	1:1000	abacm	ab194583
Anti-Bax antibody [E63]	1:1000	abacm	ab32503
Anti-Cytochrome C antibody [EPR1327]	1:1000	abcam	ab133504
Cleaved Caspase-3 (Asp175) (5A1E) Rabbit mAb	1:1000	Cell Signaling Technology	#9664
Caspase-9 (C9) Mouse mAb	1:1000	Cell Signaling Technology	#9508
Cleaved-Caspase 1 Rabbit pAb	1:500	Chengdu Zen-Bioscience	341030
IL-1 beta Rabbit pAb	1:500	Chengdu Zen-Bioscience	511369
NLRP3 Rabbit pAb	1:500	Chengdu Zen-Bioscience	381207
β-Actin Antibody	1:1000	Cell Signaling Technology	#4967

## References

[1] Bin-Jumah M., Abdel-Fattah A.M., Saied E.M., El-Seedi H.R., Abdel-Daim M.M. (2021). Acrylamide-induced peripheral neuropathy: Manifestations, mechanisms, and potential treatment modalities. Environ. Sci. Pollut. Res. Int..

[2] Peivasteh-Roudsari L., Karami M., Barzegar-Bafrouei R., Samiee S., Karami H., Tajdar-Oranj B., Mahdavi V., Alizadeh A.M., Sadighara P., Oliveri Conti G. (2024). Toxicity, metabolism, and mitigation strategies of acrylamide: A comprehensive review. Int. J. Environ. Health Res..

[3] Bušová M., Bencko V., Veszelits Laktičová K., Holcátová I., Vargová M. (2020). Risk of exposure to acrylamide. Cent. Eur. J. Public. Health.

[4] Tareke E., Rydberg P., Karlsson P., Eriksson S., Törnqvist M. (2002). Analysis of acrylamide, a carcinogen formed in heated foodstuffs. J. Agric. Food Chem..

[5] Mottram D.S., Wedzicha B.L., Dodson A.T. (2002). Acrylamide is formed in the Maillard reaction. Nature.

[6] Rasool A., Luo X., Zhang Q., Jia C., Zhao S., Liu R., Rong J., Zhou G., Wang B., Kuai J. (2025). Acrylamide and Advanced Glycation End Products in Frying Food: Formation, Effects, and Harmfulness. Foods.

[7] Rifai L., Saleh F.A. (2020). A Review on Acrylamide in Food: Occurrence, Toxicity, and Mitigation Strategies. Int. J. Toxicol..

[8] Koszucka A., Nowak A., Nowak I., Motyl I. (2020). Acrylamide in human diet, its metabolism, toxicity, inactivation and the associated European Union legal regulations in food industry. Crit. Rev. Food Sci. Nutr..

[9] Lindeman B., Johansson Y., Andreassen M., Husøy T., Dirven H., Hofer T., Knutsen H.K., Caspersen I.H., Vejrup K., Paulsen R.E. (2021). Does the food processing contaminant acrylamide cause developmental neurotoxicity? A review and identification of knowledge gaps. Reprod. Toxicol..

[10] Zhang L., Yang L., Luo Y., Dong L., Chen F. (2023). Acrylamide-Induced Hepatotoxicity Through Oxidative Stress: Mechanisms and Interventions. Antioxid. Redox Signal..

[11] Zhao M., Zhang B., Deng L. (2022). The Mechanism of Acrylamide-Induced Neurotoxicity: Current Status and Future Perspectives. Front. Nutr..

[12] Teleanu D.M., Niculescu A.G., Lungu I.I., Radu C.I., Vladâcenco O., Roza E., Costăchescu B., Grumezescu A.M., Teleanu R.I. (2022). An Overview of Oxidative Stress, Neuroinflammation, and Neurodegenerative Diseases. Int. J. Mol. Sci..

[13] Aroor A.R., Mandavia C., Ren J., Sowers J.R., Pulakat L. (2012). Mitochondria and Oxidative Stress in the Cardiorenal Metabolic Syndrome. Cardiorenal Med..

[14] Liu Z., Song G., Zou C., Liu G., Wu W., Yuan T., Liu X. (2015). Acrylamide induces mitochondrial dysfunction and apoptosis in BV-2 microglial cells. Free Radic. Biol. Med..

[15] Chen J.H., Yang C.H., Wang Y.S., Lee J.G., Cheng C.H., Chou C.C. (2013). Acrylamide-induced mitochondria collapse and apoptosis in human astrocytoma cells. Food Chem. Toxicol..

[16] Yang L., Dong L., Zhang L., Bai J., Chen F., Luo Y. (2021). Acrylamide Induces Abnormal mtDNA Expression by Causing Mitochondrial ROS Accumulation, Biogenesis, and Dynamics Disorders. J. Agric. Food Chem..

[17] Guo J., Cao X., Hu X., Li S., Wang J. (2020). The anti-apoptotic, antioxidant and anti-inflammatory effects of curcumin on acrylamide-induced neurotoxicity in rats. BMC Pharmacol. Toxicol..

[18] Mattioli R., Francioso A., Mosca L., Silva P. (2020). Anthocyanins: A Comprehensive Review of Their Chemical Properties and Health Effects on Cardiovascular and Neurodegenerative Diseases. Molecules.

[19] Victoria-Campos C.I., Ornelas-Paz J.J., Rios-Velasco C., Ruiz-Cruz S., Ornelas-Paz J., Del Toro-Sánchez C.L., Márquez-Ríos E., Calderón-Loera R. (2024). Relevance of Anthocyanin Metabolites Generated During Digestion on Bioactivity Attributed to Intact Anthocyanins. Foods.

[20] Sadowska-Bartosz I., Bartosz G. (2024). Antioxidant Activity of Anthocyanins and Anthocyanidins: A Critical Review. Int. J. Mol. Sci..

[21] Talavera S., Felgines C., Texier O., Besson C., Gil-Izquierdo A., Lamaison J.L. (2005). Remesy C(2005) Anthocyanin metabolism in rats their distribution to digestive area, kidney, and brain. J. Agric. Food Chem..

[22] Chen Y., Chen H., Zhang W., Ding Y., Zhao T., Zhang M., Mao G., Feng W., Wu X., Yang L. (2019). Bioaccessibility and biotransformation of anthocyanin monomers following in vitro simulated gastric-intestinal digestion and in vivo metabolism in rats. Food Funct..

[23] Olivas-Aguirre F.J., Rodrigo-García J., Martínez-Ruiz N.D., Cárdenas-Robles A.I., Mendoza-Díaz S.O., Álvarez-Parrilla E., González-Aguilar G.A., de la Rosa L.A., Ramos-Jiménez A., Wall-Medrano A. (2016). Cyanidin-3-O-glucoside: Physical-Chemistry, Foodomics and Health Effects. Molecules.

[24] Oumeddour D.Z., Al-Dalali S., Zhao L., Zhao L., Wang C. (2024). Recent advances on cyanidin-3-O-glucoside in preventing obesity-related metabolic disorders: A comprehensive review. Biochem. Biophys. Res. Commun..

[25] Molonia M.S., Occhiuto C., Muscarà C., Speciale A., Bashllari R., Villarroya F., Saija A., Cimino F., Cristani M. (2020). Cyanidin-3-O-glucoside restores insulin signaling and reduces inflammation in hypertrophic adipocytes. Arch. Biochem. Biophys..

[26] Zhang J., Wu J., Liu F., Tong L., Chen Z., Chen J., He H., Xu R., Ma Y., Huang C. (2019). Neuroprotective effects of anthocyanins and its major component cyanidin-3-O-glucoside (C3G) in the central nervous system: An outlined review. Eur. J. Pharmacol..

[27] Baek H., Sanjay Park M., Lee H.J. (2023). Cyanidin-3-O-glucoside protects the brain and improves cognitive function in APPswe/PS1ΔE9 transgenic mice model. J. Neuroinflammation.

[28] Song J., Zhao M., Liu X., Zhu Y., Hu X., Chen F. (2013). Protection of cyanidin-3-glucoside against oxidative stress induced by acrylamide in human MDA-MB-231 cells. Food Chem. Toxicol..

[29] Verkhratsky A., Semyanov A. (2023). Astrocytes in Ageing. Subcell. Biochem..

[30] Santiago-Balmaseda A., Aguirre-Orozco A., Valenzuela-Arzeta I.E., Villegas-Rojas M.M., Pérez-Segura I., Jiménez-Barrios N., Hurtado-Robles E., Rodríguez-Hernández L.D., Rivera-German E.R., Guerra-Crespo M. (2024). Neurodegenerative Diseases: Unraveling the Heterogeneity of Astrocytes. Cells.

[31] Fang Z., Luo Y., Ma C., Dong L., Chen F. (2022). Blueberry Anthocyanins Extract Attenuates Acrylamide-Induced Oxidative Stress and Neuroinflammation in Rats. Oxid. Med. Cell Longev..

[32] Wang P., Ji R., Ji J., Chen F. (2019). Changes of metabolites of acrylamide and glycidamide in acrylamide-exposed rats pretreated with blueberry anthocyanins extract. Food Chem..

[33] Sadatomi D., Nakashioya K., Mamiya S., Honda S., Kameyama Y., Yamamura Y., Tanimura S., Takeda K. (2017). Mitochondrial function is required for extracellular ATP-induced NLRP3 inflammasome activation. J. Biochem..

[34] Yang L., Dong L., Zhang L., Li D., Luo Y., Chen F. (2023). Acrylamide-induced autophagy-lysosomal pathway dysfunction contributing to neurotoxicity through targeting transcription factor EB. Food Front..

[35] Sun J., Li M., Zou F., Bai S., Jiang X., Tian L., Ou S., Jiao R., Bai W. (2018). Protection of cyanidin-3-O-glucoside against acrylamide- and glycidamide-induced reproductive toxicity in leydig cells. Food Chem. Toxicol..

[36] Zhao M., Zhang B., Deng L., Zhao L. (2023). Acrylamide Induces Neurotoxicity in SH-SY5Y Cells via NLRP3-mediated Pyroptosis. Mol. Neurobiol..

[37] Yan D., Yao J., Liu Y., Zhang X., Wang Y., Chen X., Liu L., Shi N., Yan H. (2018). Tau hyperphosphorylation and P-CREB reduction are involved in acrylamide-induced spatial memory impairment: Suppression by curcumin. Brain Behav. Immun..

[38] Dag Y., Yildirim S., Sengul E., Aykurt F., Gok M., Cinar A. (2025). Therapeutic role of melatonin on acrylamide-induced neurotoxicity via reducing ER stress, inflammation, and apoptosis in a rat model. BMC Pharmacol. Toxicol..

[39] Saleh D.O., Baraka S.M., Jaleel G.A.A., Hassan A., Ahmed-Farid O.A. (2024). Eugenol alleviates acrylamide-induced rat testicular toxicity by modulating AMPK/p-AKT/mTOR signaling pathway and blood-testis barrier remodeling. Sci. Rep..

[40] Famurewa A.C., Elsawy H., Sedky A. (2024). Thymoquinone Abrogates Acrylamide-Induced Cerebellar Toxicity via Modulation of Nuclear Factor Erythroid 2-Related Factor 2/Nuclear Factor Kappa B Signaling, Oxidative Neuroinflammation, and Neuroapoptosis in Rats. J. Med. Food.

[41] Yan D., Pan X., Yao J., Wang D., Wu X., Chen X., Shi N., Yan H. (2019). MAPKs and NF-KB-Mediated Acrylamide-Induced Neuropathy in Rat Striatum and Human Neuroblastoma Cells SY5Y. J. Cell Biochem..

[42] BoespÓug E.L., Eliassen J.C., Dudley J.A., Shidler M.D., Kalt W., Summer S.S., Stein A.L., Stover A.N., Krikorian R. (2018). Enhanced neural activation with blueberry supplementation in mild cognitive impairment. Nutr. Neurosci..

[43] Suresh S., Vellapandian C. (2024). Cyanidin improves spatial memory and cognition in bisphenol A-induced rat model of Alzheimer’s-like neuropathology by restoring canonical Wnt signaling. Toxicol. Appl. Pharmacol..

[44] Zhang J., Shao X., Zhao B., Zhai L., Liu N., Gong F., Ma X., Pan X., Zhao B., Yuan Z. (2020). Neurotoxicity of perfluorooctanoic acid and post-exposure recovery due to blueberry anthocyanins in the planarians Dugesia japonica. Environ. Pollut..

[45] Gilani S.J., Bin-Jumah M.N., Al-Abbasi F.A., Imam S.S., Alshehri S., Ghoneim M.M., Shahid Nadeem M., Afzal M., Alzarea S.I., Sayyed N. (2022). Antiamnesic Potential of Malvidin on Aluminum Chloride Activated by the Free Radical Scavenging Property. ACS Omega.

[46] Lee J.S., Kim Y.R., Song I.G., Ha S.J., Kim Y.E., Baek N.I., Hong E.K. (2015). Cyanidin-3-glucoside isolated from mulberry fruit protects pancreatic β-cells against oxidative stress-induced apoptosis. Int. J. Mol. Med..

[47] Ke Z., Liu Y., Wang X., Fan Z., Chen G., Xu M., Bower K.A., Frank J.A., Ou X., Shi X. (2011). Cyanidin-3-glucoside ameliorates ethanol neurotoxicity in the developing brain. J. Neurosci. Res..

[48] Zhang Z., Yuan X., Zhao Z., Liu Y., Zhou Y., Zhu Z. (2025). Cyanidin-3-O-glucoside attenuates LPS-induced endometritis in mice via regulating PPARγ activation. Nat. Prod. Res..

[49] Jia Y., Wu C., Kim Y.S., Yang S.O., Kim Y., Kim J.S., Jeong M.Y., Lee J.H., Kim B., Lee S. (2020). A dietary anthocyanin cyanidin-3-O-glucoside binds to PPARs to regulate glucose metabolism and insulin sensitivity in mice. Commun. Biol..

[50] Lyu Q., Deng H., Wang S., El-Seedi H., Cao H., Chen L., Teng H. (2023). Dietary supplementation with casein/cyanidin-3-O-glucoside nanoparticles alters the gut microbiota in high-fat fed C57BL/6 mice. Food Chem..

[51] Huang F., Zhao R., Xia M., Shen G.X. (2020). Impact of Cyanidin-3-Glucoside on Gut Microbiota and Relationship with Metabolism and Inflammation in High Fat-High Sucrose Diet-Induced Insulin Resistant Mice. Microorganisms.

[52] Zhu L., Cao F., Hu Z., Zhou Y., Guo T., Yan S., Xie Q., Xia X., Yuan H., Li G. (2024). Cyanidin-3-O-Glucoside Alleviates Alcoholic Liver Injury via Modulating Gut Microbiota and Metabolites in Mice. Nutrients.

[53] Klemmensen M.M., Borrowman S.H., Pearce C., Pyles B., Chandra B. (2024). Mitochondrial dysfunction in neurodegenerative disorders. Neurotherapeutics.

[54] Zhang L., Dong L., Yang L., Luo Y., Chen F. (2022). MiR-27a-5p regulates acrylamide-induced mitochondrial dysfunction and intrinsic apoptosis via targeting Btf3 in rats. Food Chem..

[55] Liu L., Li Y., Chen G., Chen Q. (2023). Crosstalk between mitochondrial biogenesis and mitophagy to maintain mitochondrial homeostasis. J. Biomed. Sci..

[56] Scarpulla R.C. (2008). Transcriptional paradigms in mammalian mitochondrial biogenesis and function. Physiol. Rev..

[57] Fontecha-Barriuso M., Martin-Sanchez D., Martinez-Moreno J.M., Monsalve M., Ramos A.M., Sanchez-Niño M.D., Ruiz-Ortega M., Ortiz A., Sanz A.B. (2020). The Role of PGC-1α and Mitochondrial Biogenesis in Kidney Diseases. Biomolecules.

[58] Chan D.C. (2006). Mitochondrial fusion and fission in mammals. Annu. Rev. Cell Dev. Biol..

[59] Chen H., Chomyn A., Chan D.C. (2005). Disruption of fusion results in mitochondrial heterogeneity and dysfunction. J Biol. Chem..

[60] Griparic L., van der Wel N.N., Orozco I.J., Peters P.J., van der Bliek A.M. (2004). Loss of the intermembrane space protein Mgm1/OPA1 induces swelling and localized constrictions along the lengths of mitochondria. J. Biol. Chem..

[61] Lee Y.J., Jeong S.Y., Karbowski M., Smith C.L., Youle R.J. (2004). Roles of the mammalian mitochondrial fission and fusion mediators Fis1, Drp1, and Opa1 in apoptosis. Mol. Biol. Cell..

[62] Qiang Y., Song M., Wang S., Liu Z., Shan S., Sun Y., Ni W., Chao S., Liu Z., Zhao X. (2024). High-fat diet exacerbated motor dysfunction via necroptosis and neuroinflammation in acrylamide-induced neurotoxicity in mice. Ecotoxicol. Environ. Saf..

[63] Grel H., Woznica D., Ratajczak K., Kalwarczyk E., Anchimowicz J., Switlik W., Olejnik P., Zielonka P., Stobiecka M., Jakiela S. (2023). Mitochondrial Dynamics in Neurodegenerative Diseases: Unraveling the Role of Fusion and Fission Processes. Int. J. Mol. Sci..

[64] Hou Y., Wang Q., Han B., Chen Y., Qiao X., Wang L. (2021). CD36 promotes NLRP3 inflammasome activation via the mtROS pathway in renal tubular epithelial cells of diabetic kidneys. Cell Death Dis..

[65] Zhao M., Deng L., Lu X., Fan L., Zhu Y., Zhao L. (2022). The involvement of oxidative stress, neuronal lesions, neurotransmission impairment, and neuroinflammation in acrylamide-induced neurotoxicity in C57/BL6 mice. Environ. Sci. Pollut. Res. Int..

